# Supplementary material for: NT-proBNP serves as a prognostic marker for adverse outcomes in severe immune checkpoint inhibitor-associated myocarditis
Source: PeerJ. 2025 Oct 6;13:e20020. doi: 10.7717/peerj.20020 (PMC12510244; doi:10.7717/peerj.20020)
Supplement: Supplemental Information 1 [file peerj-13-20020-s001.docx]

**Supplemental Table S1**

Baseline characteristics of all patients with ICI-associated myocarditis

| characteristics | patients (n=71) |
| --- | --- |
| Age (＞65) | 40 (56.3) |
| Male | 50 (70.4) |
| Risk factors related to myocarditis |  |
| Smoking | 30 (42.3) |
| Hypertension | 17 (23.9) |
| DM | 11 (15.5) |
| CHD | 5 (7.0) |
| Others | 3 (4.2) |
| Cancer type, n (%) |  |
| Lung cancer | 29 (40.9) |
| Gastric cancer | 10 (14.1) |
| Esophageal cancer | 8 (11.3) |
| Thymic Carcinoma | 5 (7.0) |
| Liver Cancer | 4 (5.6) |
| Cholangiocarcinoma | 3 (4.2) |
| Bladder Cancer | 3 (4.2) |
| Gallbladder cancer | 2 (2.8) |
| Ampullary adenocarcinoma | 1 (1.4) |
| Colorectal cancer | 1 (1.4) |
| Melanoma | 1 (1.4) |
| Renal cell carcinoma | 1 (1.4) |
| Breast cancer | 1 (1.4) |
| Cervical cancer | 1 (1.4) |
| Nasopharyngeal carcinoma | 1 (1.4) |
| Symptoms of myocarditis |  |
| Dyspnea | 25 (35.2) |
| Palpitations | 16 (22.5) |
| Chest pain | 10 (14.1) |
| Muscle weakness | 6 (8.5) |
| Lightheadedness | 4 (5.6) |
| Abdominal pain | 1 (1.4) |
| Asymptomatic | 9 (12.7) |
| Multi-organ irAEs |  |
| Myositis | 13 (31.0) |
| Thyroiditis | 10 (23.7) |
| Hepatitis | 6 (14.3) |
| Pneumonitis | 5 (11.9) |
| IM3OS | 3 (7.1) |
| Dermatitis | 2 (4.8) |
| Nephritis | 1 (2.4) |
| Pancreatitis | 1 (2.4) |
| hypophysitis | 1 (2.4) |
| Grade of myocarditis |  |
| 1 | 10 (14.1) |
| 2 | 28 (39.4) |
| 3 | 13 (18.3) |
| 4 | 20 (28.2) |
| Line of Therapy |  |
| First line | 41 (57.7) |
| Second line | 19 (26.8) |
| Third line and beyond | 11 (15.5) |
| Immunotherapy Drugs |  |
| Sintilimab | 14 (19.6) |
| Camrelizumab | 14 (19.6) |
| Pembrolizumab | 9 (12.7) |
| Tislelizumab | 9 (12.7) |
| Toripalimab | 6 (8.6) |
| Durvalumab | 4 (5.6) |
| Nivolumab | 3 (4.2) |
| Serplulimab | 3 (4.2) |
| Envafolimab | 3 (4.2) |
| Atezolizumab | 2 (2.8) |
| Penpulimab | 1 (1.4) |
| lvonescimab | 1 (1.4) |
| Candonilimab | 1 (1.4) |
| Socazolimab | 1 (1.4) |

Data are presented as n (%). Abbreviations: DM, diabetes mellitus; CHD, coronary heart disease; BMI, body mass index; irAEs, immune-related adverse events; IM3OS, myocarditis concomitant with myositis and/or myasthenia gravis.


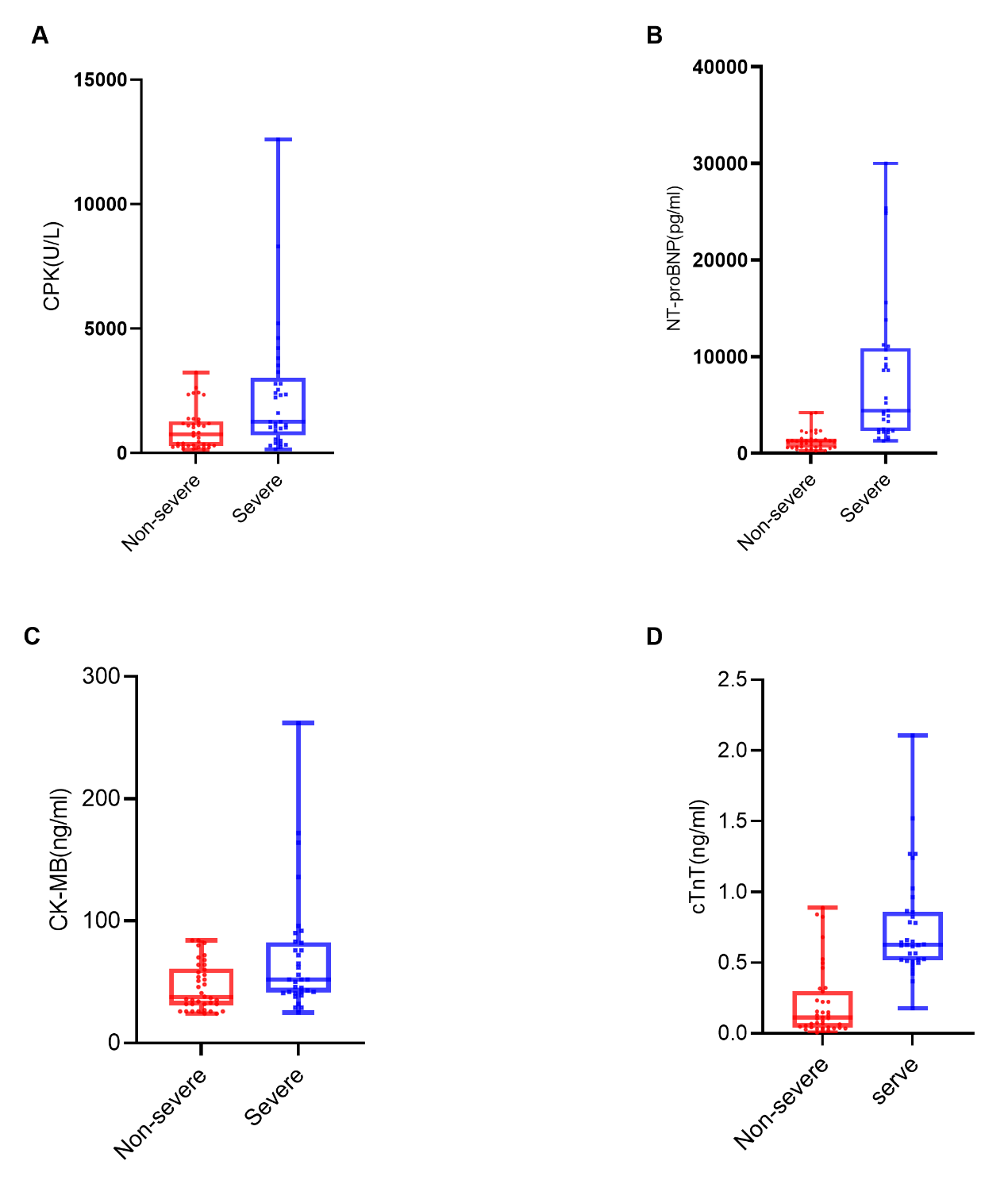


Supplementary Figure 1. Comparison of cardiac biomarker levels between patients with severe and non-severe myocarditis. (A) Creatine phosphokinase (CPK) levels (U/L) in the non-severe and severe myocarditis groups. (B) N-terminal pro-brain natriuretic peptide (NT-proBNP) levels (pg/ml) in the non-severe and severe myocarditis groups. (C) Creatine kinase-MB (CK-MB) levels (ng/ml) in the non-severe and severe myocarditis groups. (D) cardiac troponin T (cTnT) levels(ng/ml) in the non-severe and severe myocarditis groups. The median is indicated by the central line, the box edges represent the first and third quartiles (IQR), and the whiskers extend to the most extreme data points that are not considered outliers. All comparisons revealed statistically significant differences (P < 0.001).
