# Supplementary material for: NT-proBNP serves as a prognostic marker for adverse outcomes in severe immune checkpoint inhibitor-associated myocarditis
Source: PeerJ. 2025 Oct 6;13:e20020. doi: 10.7717/peerj.20020 (PMC12510244; doi:10.7717/peerj.20020)
Supplement: Supplemental Information 3 [file peerj-13-20020-s003.docx]

STROBE Statement—checklist of items that should be included in reports of observational studies

|  | Item No. | Recommendation | Page  No. | Relevant text from manuscript |
| --- | --- | --- | --- | --- |
| **Title and abstract** | 1 | (*a*) Indicate the study’s design with a commonly used term in the title or the abstract | 1 | This single-center retrospective cohort study analyzed 71 patients |
|  |  | (*b*) Provide in the abstract an informative and balanced summary of what was done and what was found | 1 | Severe myocarditis (Grades 3–4) occurred in 33 patients (46.5%), with an overall mortality rate of 54.5%. NT-proBNP levels were significantly elevated in fatal cases versus survivors... |
| Introduction | | | |  |
| Background/rationale | 2 | Explain the scientific background and rationale for the investigation being reported | 2 | Despite remarkable advances in cancer therapy with ICIs, their broader success is limited by immune-related adverse events (irAEs)... |
| Objectives | 3 | State specific objectives, including any prespecified hypotheses | 2 | We conducted a retrospective analysis of 71 patients diagnosed with ICI-associated myocarditis to characterize its clinical features and treatment outcomes, … |
| Methods | | | |  |
| Study design | 4 | Present key elements of study design early in the paper | 3 | This retrospective cohort study included consecutive patients diagnosed with ICI-associated myocarditis following immune checkpoint inhibitor (ICI) therapy... |
| Setting | 5 | Describe the setting, locations, and relevant dates, including periods of recruitment, exposure, follow-up, and data collection | 3 | between August 2018 and August 2024. Data were extracted from electronic medical records and included demographics… |
| Participants | 6 | (*a*) *Cohort study*—Give the eligibility criteria, and the sources and methods of selection of participants. Describe methods of follow-up  *Case-control study*—Give the eligibility criteria, and the sources and methods of case ascertainment and control selection. Give the rationale for the choice of cases and controls  *Cross-sectional study*—Give the eligibility criteria, and the sources and methods of selection of participants | 3 | Inclusion criteria comprised: (1) confirmed ICI administration; (2) elevated cardiac biomarkers (troponin T or NT-proBNP) with or without… |
|  |  | (*b*) *Cohort study*—For matched studies, give matching criteria and number of exposed and unexposed  *Case-control study*—For matched studies, give matching criteria and the number of controls per case | / | / |
| Variables | 7 | Clearly define all outcomes, exposures, predictors, potential confounders, and effect modifiers. Give diagnostic criteria, if applicable | 3-4 | Cardiac biomarkers, including NT-proBNP and troponin T, were assessed... The severity of ICI-associated myocarditis, according to the American Society of Clinical Oncology (ASCO) Clinical Practice Guideline, is defined as... |
| Data sources/ measurement | 8* | For each variable of interest, give sources of data and details of methods of assessment (measurement). Describe comparability of assessment methods if there is more than one group | 3-4 | Data were extracted from electronic medical records and included demographics (age, sex), medical history (smoking status, comorbidities), clinical manifestations (symptoms, signs), laboratory results (cardiac troponin T, NT-proBNP), diagnostic imaging (ECG, echocardiography)... All comparisons were statistically significant (P < 0.001) |
| Bias | 9 | Describe any efforts to address potential sources of bias | 4-5 | Exclusion criteria included insufficient diagnostic evidence…; Binary logistic regression analyses were conducted in R (v4.4.2) using the logistf package with Firth's bias-reduced penalized likelihood method to address potential separation issues in small sample sizes |
| Study size | 10 | Explain how the study size was arrived at | 3 | Between August 2018 and August 2024, 7,157 cancer patients received ICI therapy. Among these, 91 individuals were clinically suspected to have ICI-associated myocarditis. …Finally, 71 patients diagnosed with ICI-associated myocarditis were included in this study |

Continued on next page

| Quantitative variables | 11 | Explain how quantitative variables were handled in the analyses. If applicable, describe which groupings were chosen and why | 4-5 | Continuous non-normally distributed variables (e.g., biomarker levels) were reported as median with interquartile range (IQR) and compared using Mann-Whitney U tests in GraphPad Prism |
| --- | --- | --- | --- | --- |
| Statistical methods | 12 | (*a*) Describe all statistical methods, including those used to control for confounding | 4-5 | All statistical analyses and graphical representations were performed using GraphPad Prism 9.0 (GraphPad Software, Inc., La Jolla, CA, USA) and R version 4.4.2 (R Foundation for Statistical Computing, Vienna, Austria). Continuous non-normally distributed variables… |
|  |  | (*b*) Describe any methods used to examine subgroups and interactions | 4-5 | Binary logistic regression analysis, incorporating NT-proBNP, cardiac troponin T (cTnT), creatine phosphokinase (CPK), creatine kinase MB isoenzyme (CK-MB) and age (≤65 years vs. >65 years), revealed that elevated NT-proBNP levels are an independent predictor of adverse outcomes in patients with severe myocarditis |
|  |  | (*c*) Explain how missing data were addressed | / | / |
|  |  | (*d*) *Cohort study*—If applicable, explain how loss to follow-up was addressed  *Case-control study*—If applicable, explain how matching of cases and controls was addressed  *Cross-sectional study*—If applicable, describe analytical methods taking account of sampling strategy | / | / |
|  |  | (*e*) Describe any sensitivity analyses | / | / |
| Results | | | | |
| Participants | 13* | (a) Report numbers of individuals at each stage of study—eg numbers potentially eligible, examined for eligibility, confirmed eligible, included in the study, completing follow-up, and analysed | 4 | Between August 2018 and August 2024, 7,157 cancer patients received ICI therapy. Among these, 91 individuals were clinically suspected to have ICI-associated myocarditis. Following MDT deliberation, 12 cases… |
|  |  | (b) Give reasons for non-participation at each stage | 4 | Following MDT deliberation, 12 cases were excluded because of alternative explanations for their elevated cardiac biomarkers or chest discomfort. Additionally, 8 patients were excluded because of incomplete information |
|  |  | (c) Consider use of a flow diagram | 4 | Finally, 71 patients diagnosed with ICI-associated myocarditis were included in this study (Fig. 1) |
| Descriptive data | 14* | (a) Give characteristics of study participants (eg demographic, clinical, social) and information on exposures and potential confounders | 4-5 | Among them, more than half were non-smokers. Most patients were male and over 65 years old... This study included 15 tumor types, with lung cancer being the most prevalent (40.9%), followed by gastric cancer (14.1%) and esophageal cancer (11.3%) |
|  |  | (b) Indicate number of participants with missing data for each variable of interest | 4 | Additionally, 8 patients were excluded because of incomplete information. |
|  |  | (c) *Cohort study*—Summarise follow-up time (eg, average and total amount) | 5 | Myocarditis onset occurred at a median of 50 days (IQR: 29-90 days) after the initiation of immune checkpoint inhibitor therapy… |
| Outcome data | 15* | *Cohort study*—Report numbers of outcome events or summary measures over time | 5-6 | Severe myocarditis (Grades 3–4) occurred in 33 patients (46.5%), with an overall mortality rate of 54.5% |
|  |  | *Case-control study—*Report numbers in each exposure category, or summary measures of exposure | */* | */* |
|  |  | *Cross-sectional study—*Report numbers of outcome events or summary measures | */* | */* |
| Main results | 16 | (*a*) Give unadjusted estimates and, if applicable, confounder-adjusted estimates and their precision (eg, 95% confidence interval). Make clear which confounders were adjusted for and why they were included | 6 | Binary logistic regression analysis, incorporating NT-proBNP, cardiac troponin T (cTnT), creatine phosphokinase (CPK), creatine kinase MB isoenzyme (CK-MB) and age (≤65 years vs. >65 years), revealed that elevated NT-proBNP levels (OR = 4.33, 95% CI [1.21, 21.94], P = 0.023) are an independent predictor of adverse outcomes in patients with severe myocarditis |
|  |  | (*b*) Report category boundaries when continuous variables were categorized | 6 | NT-proBNP levels were significantly elevated in fatal cases versus survivors (median: 13,804 vs. 4,050 pg/mL; P < 0.001) |
|  |  | (*c*) If relevant, consider translating estimates of relative risk into absolute risk for a meaningful time period | / | / |

Continued on next page

| Other analyses | 17 | Report other analyses done—eg analyses of subgroups and interactions, and sensitivity analyses | 6-7 | Among nine patients rechallenged with immunotherapy, seven (77.8%) tolerated subsequent cycles without recurrent immune toxicity, while two with prior Grade 2 myocarditis experienced symptom recurrence… |
| --- | --- | --- | --- | --- |
| Discussion | | | | |
| Key results | 18 | Summarise key results with reference to study objectives | 7 | Severe myocarditis (Grades 3–4) occurred in 33 patients (46.5%), with an overall mortality rate of 54.5%. NT-proBNP levels were significantly elevated in fatal cases versus survivors… |
| Limitations | 19 | Discuss limitations of the study, taking into account sources of potential bias or imprecision. Discuss both direction and magnitude of any potential bias | 7 | Despite providing valuable insights into ICI-associated myocarditis, this study has several limitations that need to be considered when interpreting the results. First, this was a retrospective study, which may be subject to incomplete or inaccurate recorded data… |
| Interpretation | 20 | Give a cautious overall interpretation of results considering objectives, limitations, multiplicity of analyses, results from similar studies, and other relevant evidence | 7 | ICIs have significantly improved cancer outcomes but are associated with severe adverse events, notably ICI-associated myocarditis, characterized by low incidence and high mortality. Our study underscores… |
| Generalisability | 21 | Discuss the generalisability (external validity) of the study results | 7 | This study was conducted at a single center with a limited sample size, which may lack representativeness. Therefore, the generalizability of the findings may be limited and requires validation in multi-center studies with larger samples |
| Other information | |  | | |
| Funding | 22 | Give the source of funding and the role of the funders for the present study and, if applicable, for the original study on which the present article is based | / | No funding |

*Give information separately for cases and controls in case-control studies and, if applicable, for exposed and unexposed groups in cohort and cross-sectional studies.

**Note:** An Explanation and Elaboration article discusses each checklist item and gives methodological background and published examples of transparent reporting. The STROBE checklist is best used in conjunction with this article (freely available on the Web sites of PLoS Medicine at http://www.plosmedicine.org/, Annals of Internal Medicine at http://www.annals.org/, and Epidemiology at http://www.epidem.com/). Information on the STROBE Initiative is available at www.strobe-statement.org.
